# Can Infrared Thermal Imaging Reflect Exercise Load? An Incremental Cycling Exercise Study

**DOI:** 10.3390/bioengineering12030280

**Published:** 2025-03-11

**Authors:** Chenxi Hu, Ning Du, Zhongqian Liu, Yafeng Song

**Affiliations:** Department of Chinese Academy of Sport and Health, Beijing Sport University, Beijing 100084, China; hcx212324@163.com (C.H.); duning304@163.com (N.D.); lawliet2719606949@gmail.com (Z.L.)

**Keywords:** training load, infrared thermography (IRT), entropy analysis, external load, oxygen consumption, thermoregulation

## Abstract

Monitoring the training load is crucial in sports science research, as it provides scientific evidence for assessing training effects, optimizing athletic performance, and preventing overtraining by quantifying both external and internal loads. Although traditional monitoring methods have made significant progress, infrared thermography (IRT) technology, with its non-contact, real-time, and non-invasive characteristics, is gradually emerging as an effective tool for evaluating the relationship between the training load and physiological responses. This study evaluated 31 healthy male adults (age 21.9 ± 2.7 years, weight 75 ± 8.26 kg, and training duration 240 ± 65 min/week) performing incremental exhaustive exercise on a cycle ergometer (with a 60W starting load, increasing by 20W per minute). Entropy analysis was used to quantitatively assess the surface radiation patterns of regions of interest (forehead, chest, and abdomen) obtained through thermal imaging. Compared to baseline, significant differences in the surface radiation patterns of the regions of interest were observed at the point of exhaustion (*p* ≤ 0.01). Correlation analysis revealed strong associations between the external load, oxygen consumption, and chest temperature entropy (r = 0.973 and 0.980). Cluster analysis of the chest entropy, external load, and oxygen consumption showed a non-linear increasing trend in their inter-relationships. Further individual analysis demonstrated positive correlations between the percentage increase in the chest entropy and both the external load (r = 0.70–0.98) and oxygen consumption (r = 0.65–0.97). Entropy analysis offers a new approach for quantitatively assessing surface radiation patterns from infrared thermography, and reveals the coupling relationship between thermoregulation and metabolic responses during exercise.

## 1. Introduction

The exercise load is typically divided into the following two dimensions: external load and internal load [1]. External load refers to the objective physical characteristics of exercise, such as the intensity, frequency, duration, and volume. It is typically quantified accurately using tools such as GPS devices, power meters, or activity trackers [2]. The quantitative monitoring of external load provides a more intuitive assessment method, enabling the precise quantification of key parameters such as the exercise intensity and training volume. This offers valuable insights for evaluating athletes’ energy expenditure and training effectiveness [3]. Internal load reflects the physiological and psychological responses of the body to external load, primarily measured through physiological indicators such as the heart rate, blood lactate concentration, and maximal oxygen uptake, as well as subjective perception (RPE) [4]. According to the ACSM guidelines, the percentage of maximum heart rate and VO_2_max are commonly used to assess the exercise prescription intensity and training effectiveness [5]. Proper load adjustment can promote various adaptive changes in the body, such as enhancing the aerobic capacity and lactate tolerance. However, overtraining may lead to overtraining syndrome, which, especially in cases of inadequate recovery, can negatively affect athletes’ health and physical recovery [6].

Although exercise load monitoring is an important research area in sports science, several existing indicators, such as the blood lactate concentration, maximal oxygen uptake, and surface electromyography (sEMG), effectively reflect the load during exercise. Surface electromyography (sEMG) is commonly used to assess muscle activation patterns; however, its reliance on conductive gel-based electrodes can cause skin irritation, and its susceptibility to motion artifacts during dynamic movements often leads to signal distortion, compromising its reliability in sports settings [7]. Gas analysis systems remain the gold standard for measuring VO_2_max and other metabolic parameters, yet their bulky design and strict laboratory requirements significantly restrict their applicability in field-based training environments [8]. Blood lactate testing provides accurate metabolic load quantification [9]; however, its invasive nature may induce stress responses in participants, limiting its practicality for frequent assessments. Considering the practical application requirements of exercise testing and the need to reduce invasive measurements, researchers continue to explore new methods and tools for load monitoring. Infrared thermography (IRT) is a non-contact thermal detection technology based on infrared radiation principles. It works by using infrared detectors and optical imaging devices to capture infrared radiation signals from the surface temperature distribution of objects, converting them into visual thermal images and temperature data [10]. IRT technology, known for its non-contact, real-time, and non-invasive characteristics, has found widespread applications in the field of exercise science [11].

During exercise, thermoregulation is closely linked to load variations, reflecting the complexity of physiological regulation. As exercise intensity and duration increase, muscle metabolism intensifies, generating heat that raises the core body temperature, which often limits the endurance performance. To maintain thermal balance, the body regulates skin blood flow to dissipate core heat, while skin temperature (Tsk) and surface radiation temperature (Tsr) reflect changes in internal heat distribution and dissipation [12]. In dynamic exercise, skin temperature changes exhibit complex patterns. In a study on elite athletes during an incremental cycling test, as the exercise load increased, the skin temperature initially decreased and later rose. The initial decline corresponded to the mechanism of blood redistribution to working muscles, while the subsequent increase near fatigue reflected the body’s dynamic adaptation of thermoregulatory mechanisms [13]. Similar phenomena were observed in incremental running exercises, where skin temperature decreased at the beginning, with the thighs and forearms responding first. At the end of the exercise, the skin temperature was 3–5 °C lower than baseline, and, during the recovery phase, the forearms and thighs showed the earliest temperature increases, followed by a gradual rise in the overall skin temperature [14]. Additionally, Hadžić’s study demonstrated that IRT can assess exercise-induced fatigue by monitoring skin temperature changes. When the fatigued quadriceps experienced a drop in power, the skin temperature showed a significant negative correlation, providing further support for the use of IRT as a tool for monitoring exercise fatigue [15]. Multiple studies have confirmed that skin temperature during exercise, as captured through thermography, presents thermal spots or multiple hotspots. Using IRT to capture the surface radiation pattern (Psr) of these thermal spots offers promising insights into the complex interplay between muscle activity and thermoregulation during exercise [16].

In thermodynamics, entropy is a crucial indicator for describing the degree of disorder within a system, quantifying the order and complexity of spatial distribution [17]. Previous studies have demonstrated that Shannon entropy and Tsallis entropy can effectively analyze skin temperature signals, providing valuable insights into the relationship between skin temperature and vital signs. These entropy measures have been successfully applied to predict ICU mortality rates, with findings indicating significantly lower entropy values in non-survivors compared to survivors [18]. Additionally, multiscale entropy has been utilized to evaluate the effects of localized cooling on skin blood flow and to investigate the relationship between skin temperature and pressure ulcer risk, offering a novel approach to understanding thermoregulation. Building on this foundation, this study aims to employ entropy analysis to explore the dynamic patterns of skin temperature variations during exercise [19,20]. Entropy analysis not only quantitatively characterizes the matrix structural features of skin temperature Psr but also explores the potential relationship between skin temperature distribution and metabolic regulation. While previous studies have used region-of-interest (ROI) metrics, such as average temperature, temperature differences, and extreme values to assess temperature changes during exercise [21,22], these methods have been effective in evaluating temperature responses but may not fully capture the dynamic complexity of thermoregulation, particularly the spatial and temporal variations in skin temperature distribution. Despite the recognized importance of thermoregulatory responses to exercise, there is a lack of systematic research investigating the quantitative link between Psr and exercise load. While previous studies have examined skin temperature changes, they may not fully account for variations over time and across different body regions. Nonetheless, how temperature entropy evolves with exercise load and whether it can serve as a marker for physiological adaptations remain largely unexplored. The application of temperature entropy provides a new approach to analyzing skin temperature changes and their interaction with exercise load, metabolic processes, and other physiological factors.

Research has shown that the trunk and cervical regions, due to their proximity to major visceral organs, exhibit higher resting Tsk, as they have greater blood flow and higher metabolic heat production at rest [23]. Moreover, temperature variations in the central trunk region (chest and abdomen) and proximal limbs (forearms and thighs) better reflect exercise-induced blood flow redistribution and thermoregulatory mechanisms [11]. In this study, participants were required to maintain an upright seated posture and remove their upper garments to eliminate the thermal radiation measurement interference caused by clothing. The camera was positioned parallel to the frontal view of the body at a fixed distance of 80 cm, minimizing motion artifacts and ensuring temporal comparability of continuous temperature monitoring in the forehead, chest, and abdomen, which were selected as the regions of interest (ROIs) for measurement.

This study investigates the potential of temperature entropy for assessing the exercise load. By integrating infrared thermography with entropy analysis, we examined body surface temperature distribution under varying load conditions. While previous studies explored skin temperature responses, the link between exercise load and temperature distribution complexity remains unclear. To address this, we quantitatively analyze their relationship and assess the feasibility of entropy-based, non-invasive load monitoring. Through clustering analysis, we identify distinct thermoregulatory patterns, revealing inter-individual variability in load–entropy responses. We hypothesize that, as the exercise load increases, the body surface temperature Psr entropy also increases, establishing a measurable correlation. Additionally, we examine whether entropy effectively reflects exercise-induced thermoregulatory adaptations, providing a foundation for personalized training and real-time load monitoring in sports science.

## 2. Methods

### 2.1. Participants

Thirty-one male students from Beijing Sport University voluntarily participated in this study. Their mean (±SD) age, height, weight, and body mass index (BMI) were 21.9 ± 2.7 years, 178.54 ± 8.43 cm, 75.0 ± 8.26 kg, and 23.53 ± 3.41 kg/m^2^, respectively. All participants were regularly engaged in physical activities, with an average weekly training duration of 240 ± 65 min. None of the participants had a history of lower limb injuries in the past six months, cardiovascular or pulmonary diseases, or had taken medications affecting thermoregulation in the past three months. Additionally, they were instructed to abstain from alcohol and caffeine for 24 hours before testing and to remain fasted for at least 2 hours prior to the experiment.

This study was conducted in accordance with the ethical principles of the Declaration of Helsinki and received approval from the Experimental Ethics Committee of Sports Science at Beijing Sport University (Approval No. 2023153H). All participants provided informed consent and signed a written consent form.

### 2.2. Thermal Imaging

The infrared thermography device (FLIR E4, Teledyne FLIR LLC, Wilsonville, OR, USA) was used to capture thermal images in this study. This device features an infrared resolution of 60 × 80 pixels but employs FLIR Multi-Spectral Dynamic Imaging (MSX^®^) to enhance the image, producing a final output resolution of 320 × 240 pixels. It operates within a spectral range of 7.5–13 µm and has a thermal sensitivity of <0.15 °C. The emissivity was set to 0.98 to ensure accurate temperature readings [24].

**Figure 1:**
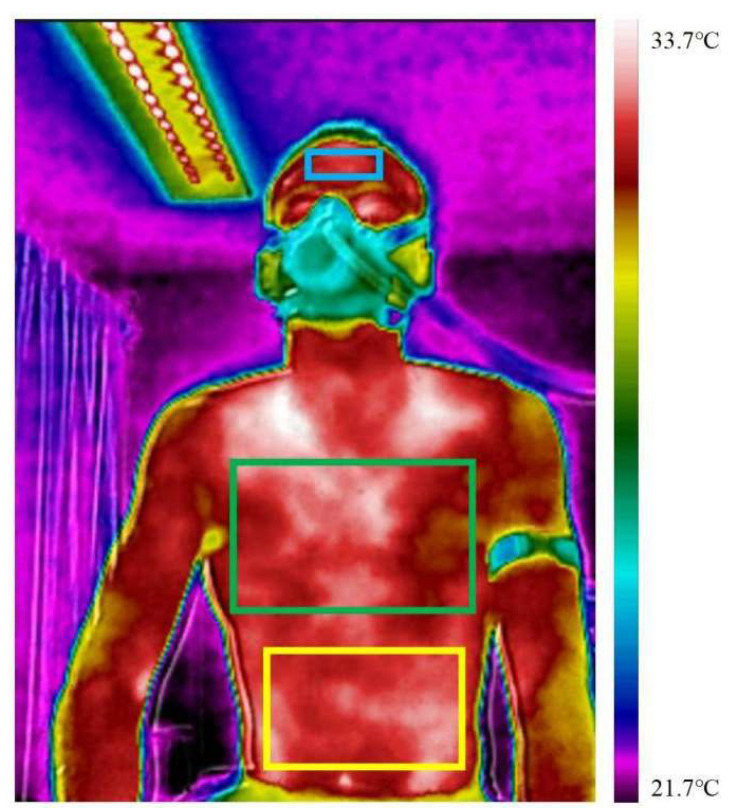
ROIs selected for the study: forehead, chest, and abdominal areas. The thermal scale bar on the right represents the surface temperature distribution, ranging from 21.7 °C to 33.7 °C. In the thermographic image, the purple and blue colors indicate cooler areas, corresponding to lower temperatures closer to the lower limit of the scale, while yellow, orange, and red represent higher temperatures, approaching the upper limit of the scale. The gradual transition between colors reflects intermediate temperature variations.

During the exercises, the laboratory maintained a controlled temperature between 20 °C and 25 °C, and a humidity level ranging from 49% to 52%, for the optimized performance of the infrared thermography device. The indoor airflow was minimal, ensuring the minimal impact of the environment on the skin temperature. Participants were required to maintain an upright posture during the entire cycling session, with the forehead, chest, and abdomen selected as the primary imaging areas (Figure 1).

To ensure consistency, the infrared thermography device was mounted on a stable frame (80 cm from the body) with the temperature sensor precisely aligned with the selected ROI. At least 2 thermal images were captured at each loading stage, and a minimum of 20 to 30 images were collected from each participant throughout the exercise session. Thermal imaging was acquired every 60 s during the resting and recovery phases, while images were taken every 30 s during the exercise period to monitor the real-time skin temperature changes.

### 2.3. Exercise Protocol

**Figure 2:**
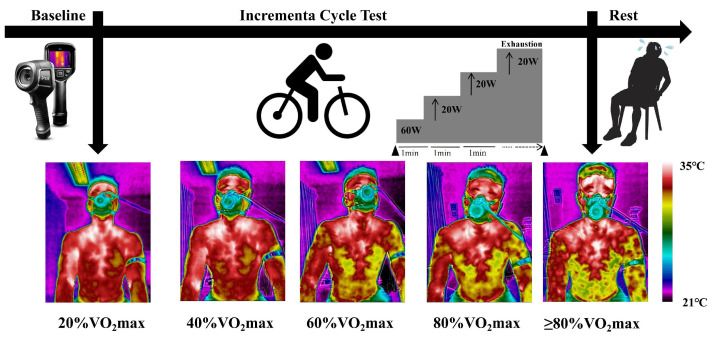
Testing protocol for measuring cycling exercise. Thermal images captured during the incremental cycle test at different exercise intensities (20%, 40%, 60%, 80%, and ≥80% VO_2_max). The thermal scale bar represents the surface temperature range from 21 °C to 35 °C, where purple indicates lower temperatures, transitioning through yellow to red, representing the highest observed temperatures.

The participants were subjected to a stepwise incremental load cycling (MONARK 893E, Monark Exercise, Vansbro, Sweden). Prior to testing, the seat height was adjusted based on individual leg length to ensure proper biomechanics and cycling efficiency. To facilitate consistent skin exposure for thermal imaging, the participants were required to remove their shirts and wear athletic shorts. After a standard warm-up of cycling on a loading of 60 W for 5 min, the participants had a 3 min rest on the bike, then performed cycling at an initial load level of 60 W while the loading was increased by 20 W per minute (Figure 2). Throughout the exercise, the participants’ heart rate (Polar Verity Sense, Polar Electro Oy, Kempele, Finland) and pulmonary ventilation were measured in real-time using the METALYZER^®^ 3B system (Cortex, Cortex Biophysik GmbH, Leipzig, Germany). A fatigue scale was used for the exhaustion evaluation and in combination with the respiratory measurements to determine the alterations to the oxygen consumption and respiratory exchange ratio (RER).

### 2.4. Feature Extraction

FLIR Tools (v6.4.17317.1002) was used to process the thermal images. After all the thermal images were imported into FLIR Tools, manual separation of the images from different ROIs was performed, and then the temperature matrices corresponding to each ROI were exported. The ROI area used for temperature matrices extraction on each thermal image for all of the participants was kept as equal as possible.

A high-dimensional dataset was obtained on the basis of the temperature matrices from all of the participants at each loading, followed by dimensionality reduction through feature extraction. The temperature matrix of each ROI was imported into R (v4.2.1) software (R Development Core Team, Vienna, Austria), rounded to an accuracy of 0.1 °C. The weighted average of the temperatures within the ROI was then calculated, with each loading level of the participant considered as a feature.

To quantify the amount of multiscale information of the selected ROI temperature matrix, the entropy was computed from the matrix. Entropy is a significant measure of information or uncertainty in a random variable. For a random variable, denoted as x, with potential outcomes x_1_, …, x_n_ occurring with respective probabilities of P(x_1_), …, P(x_n_), the Shannon entropy is defined as follows [25]:

$$H\left(x\right)=-\sum_{k=1}^{n}\;p\left(x_{k}\right)\log_{2}p\left(x_{k}\right)$$

### 2.5. Statistical Analysis

All of the entropy values and mean calculations for temperature were performed using R software (version 4.1.1, R Development Core Team, Vienna, Austria). Data visualization and statistical analyses were conducted using GraphPad Prism (version 8.0, San Diego, CA, USA) and MATLAB 2022a (MathWorks, Natick, MA, USA). Group data are presented as means (Ms) and standard deviations (SDs). The relationship between temperature entropy, exercise load, and VO_2_ was assessed using Pearson correlation analysis. To standardize the data, all entropy, VO_2_, and exercise load values were normalized using Z-scores. K-means clustering analysis was applied to identify potential patterns and trends in the data. The normality of the pre-exercise and exhaustive exercise temperature entropy of the ROIs was tested using the Shapiro–Wilk test, and, if the data did not meet normality assumptions, the Mann–Whitney U test was used to assess the differences. Statistical significance was determined based on *p*-values, with differences considered significant when *p* < 0.05. 

## 3. Results

### 3.1. Entropy Changes in ROIs During Incremental Cycling Exercise

**Figure 3:**
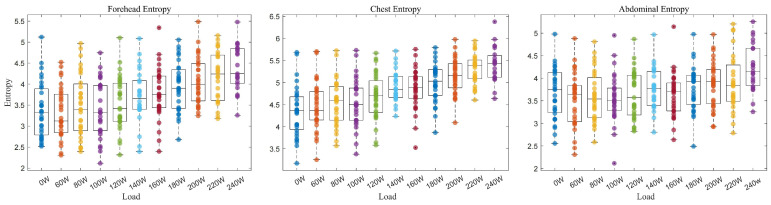
Temperature entropy of the forehead, chest, and abdomen under different loads during incremental cycling exercise.

This study analyzed the entropy changes in the forehead, chest, and abdominal temperatures under varying external load conditions during cycling exercise (0 w to 240 w) (Figure 3). Compared to pre-exercise (0 w), the Mann–Whitney U test revealed a significant increase in the forehead temperature entropy during exhaustive exercise (240 w) (3.38 vs. 4.38; *p* < 0.001). Similar results were observed for the chest (4.34 vs. 5.41; *p* < 0.001) and the abdomen (3.66 vs. 4.23; *p* = 0.01).

**Figure 4:**
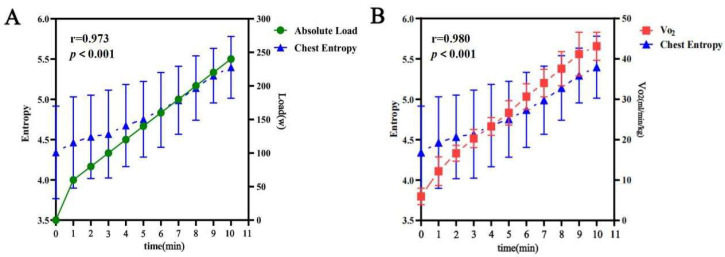
(**A**) Correlation between the absolute load and chest entropy during incremental cycling exercise. (**B**) Correlation between the oxygen consumption and chest entropy during exercise.

Further analysis revealed a strong positive correlation between the chest temperature entropy changes and absolute load (r = 0.973; *p* < 0.001). Additionally, the chest temperature entropy changes were significantly positively correlated with the oxygen consumption (r = 0.98; *p* < 0.001) (Figure 4).

### 3.2. Cluster Analysis of Absolute Load, Oxygen Consumption, and Chest Temperature Entropy

**Figure 5:**
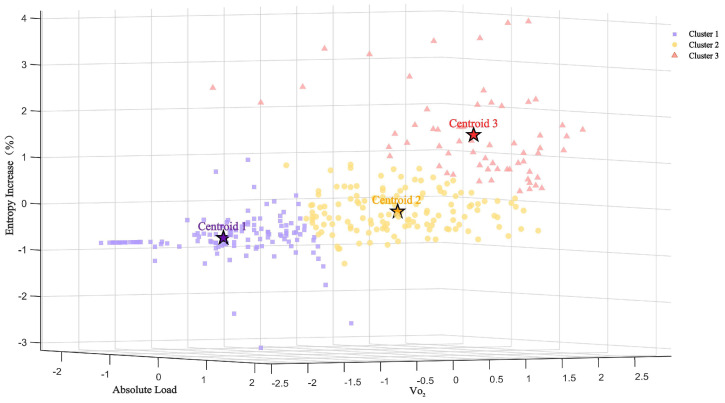
A 3D cluster plot of the absolute load, oxygen consumption (VO_2_), and chest entropy increments. Centroids 1, 2, and 3 represent the centers of the three clusters.

After standardizing all of the data using Z-scores, a 3D scatter plot was constructed based on the absolute load, oxygen consumption (VO_2_), and chest temperature entropy increments (Figure 5). The K-means clustering analysis divided the data into three clusters. The results showed that Cluster 1 was distributed in the region of the low absolute load, low-VO_2_, and low-temperature entropy increment, Cluster 2 was located in the moderate range, and Cluster 3 was concentrated in the region of high absolute load, high-VO_2_, and high-temperature entropy increment. The centroids of each cluster indicated a gradual upward trend in the temperature entropy with an increasing absolute load and VO_2_.

### 3.3. Individual Correlation Between Entropy Increase and Load

**Figure 6:**
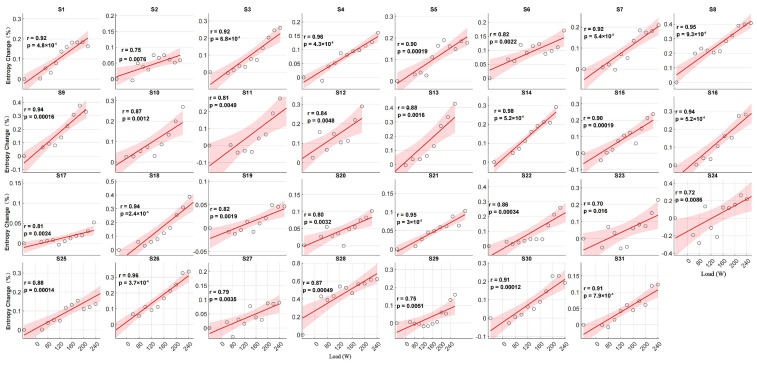
Changes in the chest entropy increments and external load during incremental exercise for 31 participants.

The correlation between the chest temperature entropy increment percentages and external load was analyzed for each participant during exercise. The results showed a significant positive correlation between the entropy increment percentages and external load for all of the participants (Pearson correlation coefficient r ranged from 0.70 to 0.98; *p* < 0.05) (Figure 6).

Specifically, for most of the participants, the entropy increment percentages of the chest exhibited a significant linear relationship with the external load. This indicates that the entropy increment percentages of the chest temperature effectively reflect the trend of the external load changes and demonstrate a certain consistency across individuals. Although the correlation for a few participants (e.g., S2 and S23) was slightly lower, it still showed a significant positive correlation.

### 3.4. Individual Correlation Analysis Between Entropy Increase and VO_2_

**Figure 7:**
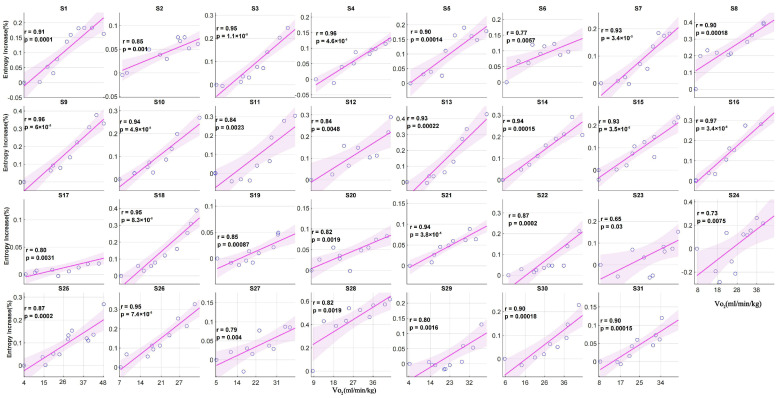
Changes in the chest entropy increments and VO_2_ during incremental exercise for 31 participants.

The correlation between the chest temperature entropy increment percentages and oxygen consumption was analyzed for each participant during exercise. The results showed a significant positive correlation between the entropy increment percentages and oxygen consumption for all of the participants (Pearson correlation coefficient r ranged from 0.65 to 0.97; *p* < 0.05) (Figure 7).

Specifically, for most of the participants, the entropy increment percentages of the chest exhibited a significant linear relationship with the internal load. This indicates that the entropy increment percentages of the chest temperature effectively reflect the trend of the internal load changes and demonstrate a certain consistency across individuals. Although the correlation for a few participants (e.g., S23 and S24) was slightly lower, it still showed a significant positive correlation.

## 4. Discussion

This study systematically investigated the effects of exercise load on the skin temperature entropy, utilizing infrared thermography and entropy analysis as a novel, non-invasive approach for load monitoring. The results demonstrated a strong correlation between the chest skin temperature entropy and external load, indicating its potential as a marker for exercise intensity assessment. Furthermore, entropy exhibited a strong correlation with oxygen consumption (VO_2_) across incremental exercise, reinforcing its role in tracking metabolic demands. Cluster analysis identified three distinct thermoregulatory response patterns, corresponding to low, moderate, and high exercise intensities, highlighting inter-individual variability. The individual case analysis of 31 participants consistently demonstrated a positive correlation between entropy and load, reinforcing its robustness as a physiological marker.

### 4.1. Entropy Analysis of ROIs Before and After Exercise

As the exercise load increases, the complexity of the skin temperature distribution significantly rises, especially in the chest region. During exercise, the body’s metabolic demands surge, leading to pronounced changes in the heat distribution complexity and entropy values. Similar findings were observed in incremental running exercises, where certain areas with elevated temperatures exhibited tree-like patterns during the baseline and recovery phases [14], which this study corroborates. Some studies suggest that temperature changes before and after exercise are associated with systemic inflammatory responses to the load, which may lead to delayed onset muscle soreness (DOMS) [26].

In graded running, cycling, and other physical activities, multiple studies have shown that post-exercise thermographic images of participants revealed localized areas of skin temperature that formed thermal spots. These spots exhibited a trend toward more discrete distribution patterns [13,27,28]. Similar findings were reported in animal studies, where horses displayed thermal spots on their skin post-exercise, often presenting irregular distribution patterns [29]. This study utilized entropy analysis to quantitatively evaluate the distribution of these thermal spots, revealing the complexity of skin temperature distribution after exercise.

### 4.2. Correlation Between Chest Entropy and Exercise Load

Studies have shown that, during incremental exercise, the forehead temperature begins to correlate positively with lactate levels when blood lactate reaches 4 mmol/L [30]. In contrast, this study found that the chest temperature entropy exhibited a significant positive correlation with both the external load and oxygen consumption throughout the exercise. Post-exercise thermographic imaging captured multi-hotspot and tree-like Psr distributions on the body, possibly representing the extensive network of perforator vessels [14]. This observation may help to explain the increase in entropy during incremental exercise.

As the exercise intensity increases, sympathetic nerve activity also intensifies, leading to the vasoconstriction of skin blood vessels and the redistribution of blood flow [31]. This process is regulated by the release of norepinephrine and neuropeptide Y from the sympathetic nervous system, which constricts cutaneous blood vessels to maintain the core temperature and supply oxygen to active muscles [32]. With increasing exercise intensity, blood redistribution becomes more pronounced, and endogenous neural mechanisms trigger reflex neurogenic vasodilation of skin blood vessels, known as the “perforator vessel” phenomenon. This occurs particularly when exercise intensity and body temperature reach certain thresholds, leading to increased skin blood flow and the visualization of tree-like vascular structures [11].

These physiological phenomena further promote an increase in entropy, as the complexity of blood circulation enhances the heterogeneity of skin temperature distribution. The dense microvascular network in the chest region plays a key role in heat regulation and blood flow distribution. The highly developed microcirculation provides abundant mechanisms for heat dissipation and retention [33], which may explain the increased entropy of chest temperature distribution. During exercise, the dynamic changes in chest blood flow are regulated not only by cardiac output but also by the response of skin microvessels to temperature variations. These microvessels play a crucial role in heat dissipation and retention, ultimately contributing to the increase in chest temperature entropy.

### 4.3. K-Means Clustering Analysis and Entropy Variation Patterns

Clustering analysis revealed that entropy changes under different load conditions not only followed an increasing trend but also demonstrated a non-linear growth. Observations from cluster centroids showed that, from low to moderate loads, the increase in entropy was relatively gradual. However, from moderate to high loads, the entropy increment became more pronounced, reflecting the sharp rise in the metabolic load. This non-linear escalation highlights the increased complexity of thermoregulatory mechanisms and the dramatic rise in metabolic demand under high-load conditions. Compared to linear models, entropy analysis appears better suited to capturing the complex, non-linear relationship between the exercise load and metabolic demand.

Damiano’s study demonstrated that, during squat exercises at different speeds, skin temperature dynamics in the legs exhibited non-steady-state characteristics with prolonged squatting. This phenomenon may be attributed to differences in vasoconstrictive responses, blood redistribution, and muscle blood flow restriction under varying loads or speeds [34]. Currently, no studies specifically address the non-linear behavior of chest entropy increments.

As the exercise load increases, during graded exercise, the body initially maintains physiological homeostasis by regulating cardiac and respiratory activity. However, when the exercise intensity exceeds the anaerobic threshold, physiological responses such as VO_2_ and blood lactate concentration will show nonlinear changes [35,36].

Particularly under high-load conditions, increased sympathetic nerve discharge, the suppression of parasympathetic activity, and blood flow redistribution enable the body to rapidly adapt to rising metabolic demands [37]. This abrupt rise reflects how the body meets the sharp changes in energy demands under high-load conditions, thereby supporting exercise performance [38].

In incremental exercise, lactate accumulation also increases non-linearly. When the body exceeds the lactate threshold, cardiovascular responses are typically observed [39]. As blood lactate levels rise sharply, the heart must adapt to the high metabolic load by increasing the cardiac output and promoting skeletal muscle blood flow to meet oxygen demands [40]. This non-linear upward trend reflects the body’s rapid response to high-load metabolic demands above the lactate threshold. The entropy variations across three load conditions may reflect the coupling relationship between thermoregulation, lactate accumulation, and cardiovascular responses during incremental exhaustive exercise.

### 4.4. Individual Differences and Entropy Variation

In the individual analysis, we found that, for most participants, there was a significant positive correlation between the entropy increment percentage and the exercise load (power and oxygen consumption), while a minority exhibited lower correlations. These individual differences may be related to variations in physiological and psychological factors. Research in exercise science has validated the role of individual differences. Studies have shown that physiological factors, such as the body fat percentage, muscle mass, cardiopulmonary fitness, and training level, can significantly influence metabolic responses and related physiological signals during exercise [41,42,43]. Furthermore, individual differences are also reflected in skin temperature changes between trained and untrained groups, with the former exhibiting greater increases in the skin temperature amplitude and rate during exercise, suggesting different levels of thermoregulatory response based on the training status [24]. These factors may explain the variation in entropy increments. Thus, in practical applications, the analysis of entropy and load through thermography should be interpreted alongside the individual’s physiological characteristics to provide personalized load monitoring solutions.

### 4.5. Limitations

In this study, we selected only three ROIs, the forehead, chest, and abdomen, which did not cover the entire skin surface. This may limit the thermal information collected. Future studies will aim to include additional regions, such as the arms and thighs, to enhance the scope of the research. It is important to acknowledge that the infrared resolution of the camera used in this study may limit the spatial precision of the skin temperature measurements, potentially affecting the accuracy of the thermal pattern analysis. Future research should consider employing higher-resolution infrared thermography to capture surface radiation patterns (Psr) during exercise, enabling a more detailed exploration of the relationship between thermoregulation and exercise load. Exploring commercially available devices with a higher resolution and sensitivity could improve the measurement precision. Another limitation is that the study included only 31 male participants aged 18~24 years. Because obtaining chest temperature images posed practical and ethical constraints for enrolling female participants, our study included only males, limiting the generalizability of these findings. Future research will explore alternative ROIs that do not require chest exposure to accommodate female participants.

### 4.6. Practical Application

In practical applications, thermography combined with entropy analysis provides a non-invasive, real-time approach for monitoring exercise load, effectively capturing the strong correlation between entropy and both external (power output) and internal (VO_2_) load. This method offers a novel alternative to traditional physiological monitoring tools, which often require direct contact or invasive sampling. Furthermore, its non-invasive and wearable-free design minimizes interference with movement. Compared to lab-based methods, such as VO_2_ and blood lactate analysis, entropy-based thermography enables practical, real-world monitoring with minimal disruption, making it well suited for use in actual sports settings.

## 5. Conclusions

This study systematically investigated the changes in skin temperature entropy during incremental exercise and its relationship with exercise load (power and oxygen consumption) using infrared thermography (IRT) combined with entropy analysis. The results indicate that chest temperature entropy increases non-linearly with rising exercise intensity and correlates strongly with both load parameters, suggesting its potential sensitivity to metabolic and thermoregulatory adjustments. These findings provide new insights into the use of entropy-based thermal analysis for assessing exercise-induced physiological responses. By capturing dynamic variations in heat distribution, entropy analysis offers a complementary perspective for understanding thermoregulatory adaptation and load progression, which may contribute to future advancements in exercise monitoring and individualized training approaches.

## Data Availability

The raw data supporting the conclusions of this article will be made available by the authors on request.

## References

[1] McLaren S.J., Macpherson T.W., Coutts A.J., Hurst C., Spears I.R., Weston M. (2018). The Relationships Between Internal and External Measures of Training Load and Intensity in Team Sports: A Meta-Analysis. Sports Med..

[2] Borresen J., Lambert M.I. (2009). The quantification of training load, the training response and the effect on performance. Sports Med..

[3] Bourdon P.C., Cardinale M., Murray A., Gastin P., Kellmann M., Varley M.C., Gabbett T.J., Coutts A.J., Burgess D.J., Gregson W. (2017). Monitoring Athlete Training Loads: Consensus Statement. Int. J. Sports Physiol. Perform..

[4] Alexiou H., Coutts A.J. (2008). A comparison of methods used for quantifying internal training load in women soccer players. Int. J. Sports Physiol. Perform..

[5] Pescatello L.S. (2014). ACSM’s Guidelines for Exercise Testing and Prescription.

[6] Grandou C., Wallace L., Impellizzeri F.M., Allen N.G., Coutts A.J. (2020). Overtraining in resistance exercise: An exploratory systematic review and methodological appraisal of the literature. Sports Med..

[7] Perpetuini D., Formenti D., Cardone D., Trecroci A., Rossi A., Di Credico A., Merati G., Alberti G., Di Baldassarre A., Merla A. (2023). Can Data-Driven Supervised Machine Learning Approaches Applied to Infrared Thermal Imaging Data Estimate Muscular Activity and Fatigue?. Sensors.

[8] Shei R.-J., Holder I.G., Oumsang A.S., Paris B.A., Paris H.L. (2022). Wearable activity trackers–advanced technology or advanced marketing?. Eur. J. Appl. Physiol..

[9] Faude O., Kindermann W., Meyer T. (2009). Lactate threshold concepts: How valid are they?. Sports Med..

[10] Lahiri B.B., Bagavathiappan S., Jayakumar T., Philip J. (2012). Medical applications of infrared thermography: A review. Infrared Phys. Technol..

[11] Hillen B., Pfirrmann D., Nägele M., Simon P. (2020). Infrared thermography in exercise physiology: The dawning of exercise radiomics. Sports Med..

[12] Flouris A., Schlader Z.J. (2015). Human behavioral thermoregulation during exercise in the heat. Scand. J. Med. Sci. Sports.

[13] Ludwig N., Trecroci A., Gargano M., Formenti D., Bosio A., Rampinini E., Alberti G. (2016). Thermography for skin temperature evaluation during dynamic exercise: A case study on an incremental maximal test in elite male cyclists. Appl. Opt..

[14] Merla A., Mattei P.A., Di Donato L., Romani G.L. (2010). Thermal imaging of cutaneous temperature modifications in runners during graded exercise. Ann. Biomed. Eng..

[15] Hadžić V., Širok B., Malneršič A., Čoh M. (2019). Can infrared thermography be used to monitor fatigue during exercise? A case study. J. Sport Health Sci..

[16] Hillen B., López D.A., Pfirrmann D., Neuberger E.W., Mertinat K., Nägele M., Schömer E., Simon P. (2023). An exploratory, intra-and interindividual comparison of the deep neural network automatically measured calf surface radiation temperature during cardiopulmonary running and cycling exercise testing: A preliminary study. J. Therm. Biol..

[17] Fuchs H.U., D’Anna M., Corni F.J. (2022). Entropy and the experience of heat. Entropy.

[18] Papaioannou V.E., Chouvarda I.G., Maglaveras N.K., Baltopoulos G.I., Pneumatikos I.A. (2013). Temperature multiscale entropy analysis: A promising marker for early prediction of mortality in septic patients. Physiol. Meas..

[19] Liao F., Yang T.D., Wu F.L., Cao C., Mohamed A., Jan Y.K. (2019). Using Multiscale Entropy to Assess the Efficacy of Local Cooling on Reactive Hyperemia in People with a Spinal Cord Injury. Entropy.

[20] Rapp M.P., Bergstrom N., Padhye N.S. (2009). Contribution of skin temperature regularity to the risk of developing pressure ulcers in nursing facility residents. Adv. Ski. Wound Care.

[21] Perpetuini D., Formenti D., Cardone D., Filippini C., Merla A. (2021). Regions of interest selection and thermal imaging data analysis in sports and exercise science: A narrative review. Physiol. Meas..

[22] Rojas-Valverde D., Tomás-Carús P., Timón R., Batalha N., Sánchez-Ureña B., Gutiérrez-Vargas R., Olcina G.J. (2021). Short-term skin temperature responses to endurance exercise: A systematic review of methods and future challenges in the use of infrared thermography. Life.

[23] Fernandes Ade A., Amorim P.R., Brito C.J., Sillero-Quintana M., Bouzas Marins J.C. (2016). Regional Skin Temperature Response to Moderate Aerobic Exercise Measured by Infrared Thermography. Asian J. Sports Med..

[24] Formenti D., Ludwig N., Gargano M., Gondola M., Dellerma N., Caumo A., Alberti G. (2013). Thermal imaging of exercise-associated skin temperature changes in trained and untrained female subjects. Ann. Biomed. Eng..

[25] Bogomilsky S., Hoffer O., Shalmon G., Scheinowitz M. (2022). Preliminary study of thermal density distribution and entropy analysis during cycling exercise stress test using infrared thermography. Sci. Rep..

[26] Stewart I.B., Moghadam P., Borg D.N., Kung T., Sikka P., Minett G.M. (2020). Thermal infrared imaging can differentiate skin temperature changes associated with intense single leg exercise, but not with delayed onset of muscle soreness. J. Sports Sci. Med..

[27] Arfaoui A., Bertucci W.M., Letellier T., Polidori G. (2014). Thermoregulation during incremental exercise in masters cycling. J. Sci. Cycl..

[28] Tanda G. (2018). Total body skin temperature of runners during treadmill exercise: A pilot study. J. Therm. Anal. Calorim..

[29] Witkowska-Piłaszewicz O., Maśko M., Domino M., Winnicka A.J. (2020). Infrared thermography correlates with lactate concentration in blood during race training in horses. Animals.

[30] Akimov E., Son’Kin V.J. (2011). Skin temperature and lactate threshold during muscle work in athletes. Hum. Physiol..

[31] Thomas G.D., Segal S.S. (2004). Neural control of muscle blood flow during exercise. J. Appl. Physiol..

[32] Hodges G.J., Kosiba W.A., Zhao K., Johnson J.M. (2008). The involvement of norepinephrine, neuropeptide Y, and nitric oxide in the cutaneous vasodilator response to local heating in humans. J. Appl. Physiol..

[33] Cracowski J.-L., Roustit M. (2020). Human skin microcirculation. Compr. Physiol..

[34] Formenti D., Ludwig N., Trecroci A., Gargano M., Michielon G., Caumo A., Alberti G. (2016). Dynamics of thermographic skin temperature response during squat exercise at two different speeds. J. Therm. Biol..

[35] Poole D.C., Rossiter H.B., Brooks G.A., Gladden L.B. (2021). The anaerobic threshold: 50+ years of controversy. J. Physiol..

[36] Raleigh C., Donne B., Fleming N. (2018). Association between different Non-Invasively Derived Thresholds with Lactate Threshold during graded incremental exercise. Int. J. Exerc. Sci..

[37] Nobrega A.C., O′ Leary D., Silva B.M., Marongiu E., Piepoli M.F., Crisafulli A. (2014). Neural regulation of cardiovascular response to exercise: Role of central command and peripheral afferents. BioMed Res. Int..

[38] Fletcher G.F., Ades P.A., Kligfield P., Arena R., Balady G.J., Bittner V.A., Coke L.A., Fleg J.L., Forman D.E., Gerber T.C. (2013). Exercise standards for testing and training: A scientific statement from the American Heart Association. Circulation.

[39] Binder R.K., Wonisch M., Corra U., Cohen-Solal A., Vanhees L., Saner H., Schmid J.-P. (2008). Methodological approach to the first and second lactate threshold in incremental cardiopulmonary exercise testing. Eur. J. Prev. Cardiol..

[40] Gladden L.B. (2000). Muscle as a consumer of lactate. Med. Sci. Sports Exerc..

[41] Bouchard C., Rankinen T. (2001). Individual differences in response to regular physical activity. Med. Sci. Sports Exerc..

[42] Crouzier M., Hug F., Dorel S., Deschamps T., Tucker K., Lacourpaille L. (2019). Do individual differences in the distribution of activation between synergist muscles reflect individual strategies?. Exp. Brain Res..

[43] Boffey D., DiPrima J.A., Kendall K.L., Hill E.C., Stout J.R., Fukuda D.H. (2023). Influence of Body Composition, Load-Velocity Profiles, and Sex-Related Differences on Army Combat Fitness Test Performance. J. Strength Cond. Res..

